# The complete pineapple (*Ananas comosus*; Bromeliaceae) varieties F153 chloroplast genome sequence

**DOI:** 10.1080/23802359.2016.1174084

**Published:** 2016-06-20

**Authors:** Jin Liu, Ying-Feng Niu, Shi-Hong Liu, Shu-Bang Ni

**Affiliations:** a Yunnan Institute of Tropical Crops, Jinghong, China;; b Key Laboratory of Dai and Southern Medicine of Xishuangbanna Dai Autonomous Prefecture, Jinghong, China;; c Yunnan Branch of Institute of Medicinal Plant Development, Chinese Academy of Medical Sciences, Jinghong, Yunnan, China

**Keywords:** Chloroplast genome, pineapple varieties F153, phylogenetic analysis

## Abstract

This study reported the complete nucleotide sequence of the pineapple varieties F153 chloroplast (cp) genome. The cpDNA was 159,521 bp in length and contained 131 genes (77 protein-encoding genes, 30 tRNA genes and four rRNA genes). The phylogenetic relationship of the pineapple varieties F153 plastid was consistent with previous report.

Pineapple is a famous tropical fruit due to its very attractive aroma and very nice flavour and has a long history of human consumption (Leal & Coppens d’Eeckenbrugge 1996). Its fruit is not only appreciated as a gastronomic pleasure but also used as a digestive aid taken between meals, as well as a meat tenderizer due to the presence of a strong protease enzyme known as bromelain. Different varieties of the Ananas species have been cultivated tropical regions of world wide (Morton 1987). The chloroplast genome sequence carries rich information for plant molecular systematics and Barcoding. While as one of the famous tropical fruits from the Bromeliaceae family of the order Poale, only MD-2 pineapple chloroplast genome was reported previously (Redwan et al. 2015).

To provide a rich genetic information and improve pineapple molecular breeding in the future, we sequenced one pineapple variety chloroplast genome. The plant sample for this species of pineapple variety F153 was collected in Yunnan Institute of Tropical Crops and the dried specimen was conserved in department of plant breeding in the institute. Total DNA of the pineapple varieties F153 was sequenced with second-generation sequencing technology (Illumina HiSeq 2000, San Diego, CA). The chloroplast genome sequence reads were assembled with bioinformatic pipeline including SOAP2 software (Li et al. 2009) and several runs of manual corrections of sequence reads. Genes encoded by this genome were annotated by import the fasta format sequence to the DOGMA (Wyman et al. 2004) and recorrected by manual. The final assembled cpDNA of pineapple varieties F153 plastid is 159,521 bp in length (GenBank accession no. KU598872). This genome also contains two inverted repeated regions (IRs) of 25,768 bp, and the large single-copy (LSC) region and small single-copy (SSC) region are 85,987 bp and 18,469 bp, respectively. To study its phylogenetic relationships with in the angiosperms, 11 complete cp genome sequences from Poales and other groups of angiosperms were download for analyses. The maximum-likelihood (ML) phylogenetic tree were constructed with RAxML (Stamatakis 2006) and bootstrapped with 1000 replicates, final resulted in a single tree with –ln *L* = 4,562,564.56 and all the nodes were supported by values of 100%. The phylogenetic relationship of the pineapple varieties F153 plastid was consistent with previous report (Redwan et al. 2015) (Figure 1).

**Figure 1.**
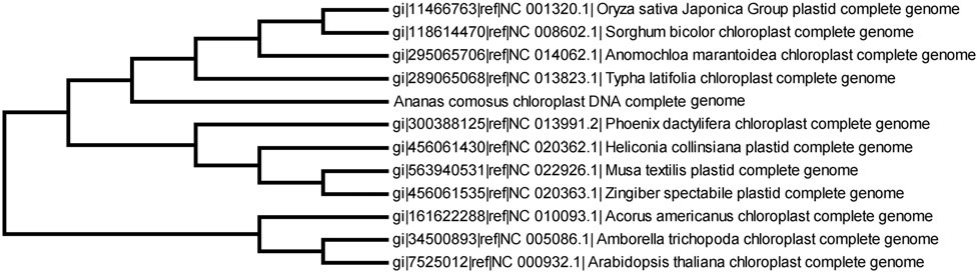
Phylogenetic tree of 12 complete chloroplast sequences including the newly sequenced *A. comosus* chloroplast with *Arabidopsis thaliana* and *Anomochloa marantoidea* as outgroups. All the nodes were supported with 100% bootstraps.
